# Standardization and validation of a novel reverse transcriptase polymerase chain reaction method for detecting virulent strains of the infectious bursal disease virus

**DOI:** 10.14202/vetworld.2024.2998-3004

**Published:** 2024-12-30

**Authors:** Vladimir Longa-Bobadilla, Phillip Ormeño-Vásquez, Manuel Criollo-Orozco, Luis Tataje-Lavanda, Katherine Huamán-Gutierrez, Ángela Montalván, Mirko Zimic, Manolo Fernández-Sanchez, Manolo Fernández-Díaz

**Affiliations:** 1 Research and Development Laboratories, FARVET S.A.C., Chincha Alta, Ica, Peru; 2 Department of Biology, Faculty of Natural Sciences and Mathematic, Federico Villarreal National University, Lima, Peru; 3 Department of Biology and Chemistry, Morehead State University, Morehead, Kentucky, USA; 4 School of Medicine, San Juan Bautista Private University, Lima, Peru; 5 Laboratories of Bioinformatics, Molecular Biology and Technology Developments, Laboratories of Research and Development, Faculty of Sciences and Philosophy, Cayetano Heredia Peruvian University, Lima, Peru

**Keywords:** alignment of sequences, infectious bursal disease virus, novel reverse transcriptase polymerase chain reaction, virulent strains, *VP5* gene

## Abstract

**Background and Aim:**

Gumboro disease is an economically crucial veterinary condition in chickens. It is caused by the infectious bursal disease virus (IBDV). This virus consists of two serotype groups, of which serotype I strain is pathogenic to chickens. For many years, the development of molecular techniques for either diagnostic purposes or surveillance of the appearance of new pathogenic strains has mainly focused on targeting the VP2 genomic region. However, due to the constant necessity for the discrimination between already prevalent vaccine strains and new pathogenic strains of this virus, it becomes imperative to have an immediate molecular method targeting a consensus sequence to achieve this task using field samples to reduce costs. Consequently, we focused on developing a novel reverse transcriptase polymerase chain reaction (RT-PCR) procedure solely for this purpose.

**Materials and Methods:**

Eight *VP5* sequences were aligned, and the sequence with the majority of nucleotide coincidences was used to design a set of consensus primers. Then, a pathogenic strain of IBDV was propagated in embryonated chicken eggs, and the viral RNA was extracted. Finally, the conditions for this novel RT-PCR were evaluated using a commercial kit and the newly designed primers.

**Results:**

After determining the optimal RT-PCR conditions, the newly designed primers successfully amplified a 402-bp consensus sequence of the *VP5* gene. In addition, these primers specifically amplified the *VP5* sequence of the IBDV-positive samples, not the other samples previously confirmed to be positive for other common poultry pathogens.

**Conclusion:**

Our novel RT-PCR procedure has been demonstrated to be helpful in selectively amplifying the consensus sequence of the *VP5* gene, indicating that this novel RT-PCR procedure constitutes an important and useful tool to execute initial discrimination of field-retrieved samples containing and not containing virulent strains of this virus before deciding to execute a blindly and more costly sequencing procedure of all the samples together.

## Introduction

Gumboro disease is caused by the infectious bursal disease virus (IBDV), which belongs to the *family Birnaviridae* and *genus* Abirnavirus. This virus can infect chickens, turkeys, ducks, guinea fowls, and ostriches. Despite this fact, the virus only causes clinical signs of this disease in chickens, and it is responsible for the high mortality in unvaccinated individuals aged between 3 and 6 weeks of age [1]. IBDV possesses a genome consisting of double-stranded RNA containing two genomic segments called A and B. In addition, two serotype groups of this virus exist, from which only serotype 1 is pathogenic to chickens and propagates inside the B cells of the bursa of Fabricius [2].

Due to these reasons, the virus is considered of great economic importance in the poultry industry. Therefore, it is imperative to develop accurate and reliable diagnostic methods to detect and quantify the virus in clinical samples. Consequently, reverse transcriptase and quantitative reverse transcriptase polymerase chain reaction methods were used, mainly targeting the*VP*2 gene [3, 4]. Nevertheless, demonstrating the field efficacy of HVT-IBD vector vaccines [5] and their posterior commercialization raises the possibility of false-positive results when using primers that target the *VP2* gene. Despite this, other molecular techniques have also been reported to target the *VP4* gene [6] or the *VP2* and *VP1* genes [7]. Moreover, when it comes to PCR procedures, their reliability as a good diagnostic tool depends mostly on good primer design and optimized PCR conditions [8], but as downsides, they require trained staff to execute them and present high associated costs to apply them in laboratory settings [9]. Thus, the most effective PCR procedures should target consensus sequences to achieve this goal because the more conserved the pathogen’s target sequence, the more effective its detection.

In this context, it was discovered that in genome segment A of IBDV, the open reading frame (ORF) A-2 region partially overlaps with the *ORF* of the IBDV polyprotein sequence, which will originate the mature VP2, VP3, and VP4 proteins. This ORF A-2 encodes a non-structural protein called VP5, which is responsible for eliciting apoptosis in host cells and possesses a sequence that is very conserved in virulent serotype I strains of this virus [10, 11].

Furthermore, surveillance for the appearance of new virulent strains constitutes a constant necessity [12, 13], as well as elucidation of their pathogenic capacity [14], and also taking into consideration that other costly molecular techniques are available to achieve this goal [7, 12, 13, 15, 16], laboratories in rural places need to have a cheaper molecular technique to execute a quick discrimination of the presence of virulent IBDV (vIBDV) and non-virulent strains in poultry samples obtained in the field. Consequently, this study developed a new reverse-transcriptase polymerase chain reaction (RT-PCR) procedure to achieve this objective.

## Materials and Methods

### Ethical approval

No ethical approval was required for the execution of this study.

### Study period and location

The study was conducted from October 2016 to July 2017 in the Laboratory of Molecular Biotechnology and Genomics of FARVET S.A.C., Chincha, Peru. This is one of the six different laboratories located in the compound of this company.

### Primer design

Eight IBDV-VP5 sequences from virulent strains submitted from different parts of the world that were available at the National Center for Biotechnology Information (NCBI) [17] were selected: MF969107.1, AF321056.1, AF321055.1, AF165151.1, JF812067.1, X16107.1, AF194428.1, and JX424076.1. Later, we copied all these sequences in FASTA format and executed an *in silico* alignment using the European Molecular Biology Laboratory (EMBL) free bioinformatics toolClustal Omega (https://www.ebi.ac.uk/Tools/msa/clustalo), with the application of the ClustalW algorithm with the character count output format parameter [18].

**Figure-1:**
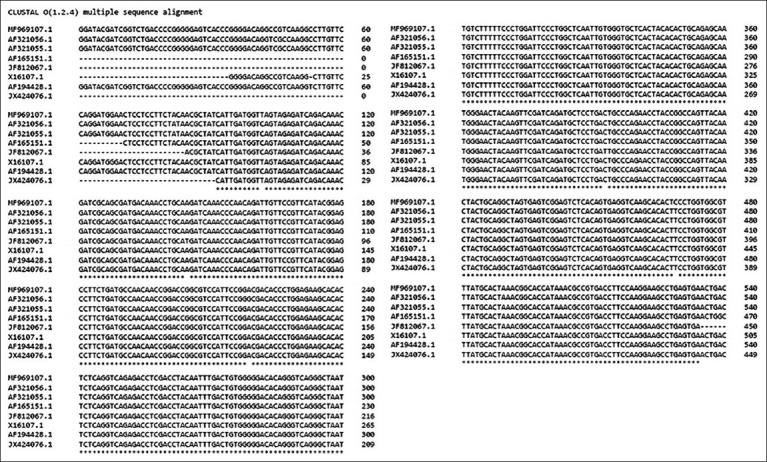
Clustal omega alignment results of the IBDV-Vp5 sequences available on GenBank (https://www.ncbi.nlm.nih.gov/genbank/). A more than 405-bp coincidence with most of the other sequences was observed (accession number AF165151.1). IBDV=Infectious bursal disease virus.

Due to most nucleotide coincidences with all other evaluated sequences (more than 405 bp), NCBI accession number AF194428.1 was selected as the template to design consensus primers for the previously mentioned region (Figure-1). Finally, we obtained the desired primer sequences using the free *in silico* bioinformatics tool Primer3 Input version 4.0 (https://primer3.ut.ee/) [19, 20].

From all the primer sequences obtained, the primers FarvetVL.VP5-F: AGCGATGACAAACCTGCAT and FarvetVL.VP5-R: CAGCGATGACAAACCTGCA were particularly selected to be evaluated due to their greater amplicon size (402 bp). A posterior *in silico* evaluation of these primers specificity was carried out using the bioinformatics tool SnapGene Viewer Version 3.2.1 (https://www.snapgene.com/snapgene-viewer) (Licensed version).

### Propagation of serotype I IBDV strain in allantoid fluid

The serotype I strain of IBDV (Farvet S.A.C.) was confirmed through a previously executed sequencing procedure (Macrogen, Korea) and was first propagated in embryonated chicken eggs. This stage consisted of the preparation of 1/5000 dilutions of each inoculum that originally presented 1 × 10^6^ viral particles of this strain of IBDV. Each dilution was then used to inoculate the chorioallantoic membrane of embryonated chicken eggs. After that, we incubated each inoculated culture for 96 h at 37°C. Finally, the total sample was harvested and stored at −20°C until the execution of total viral nucleic acid extraction.

### Viral and naïve cell nucleic RNA extraction

The total RNA from the serotype I strain of IBDV virus propagated in embryonated chicken eggs and the total RNA from naïve embryonated chicken eggs were extracted using QIAamp MinElute Virus Spin Kit (Qiagen, Hilden, Germany) and a Rneasy Kit (Qiagen, Hilden, Germany), respectively, in an automated manner with the aid of an automated device named QIAcube (Qiagen, Hilden, Germany) following manufacturer’s instructions. Furthermore, total DNA and/or RNA from samples containing common poultry pathogens were also extracted using the DNeasy Blood and Tissue Kit (Qiagen) or QIAamp MinElute Virus Spin Kit (also in an automated way and following the manufacturer’s instructions) (Qiagen). Finally, the total extracted DNA and RNA from all previously obtained samples were quantified using the Invitrogen Fluorometer Qubit 9V (Thermo Fisher Scientific, Waltham, USA) with either the Qubit dsDNA HS Assay Kit (Thermo Fisher Scientific) or theQubit RNA HS Assay Kit (Thermo Fisher Scientific), respectively, following manufacturers’ instructions.

### Preparation of serial dilutions of IBDV genomic RNA and calculation of genomic copy numbers per dilution

After quantification of IBDV total extracted RNA (10 ng), we executed serial dilutions of this sample using PCR Grade Water (Sigma-Aldrich, USA), obtaining the following dilutions: 1 ng, 100 pg, 10 pg, 1 pg, 100 ng, 10 ng, 1 ng, 100 pg, 10 pg, 1 pg, 100 fg, 10 fg, 1 fg, 100 ag, 10 ag, and 1 ag. Then, we used these dilutions for the following RT-PCR evaluations of optimal conditions, as suggested in a previous publication [21].

Finally, we calculated the theoretical IBDV genome copy number of the original sample and each dilution using the free bioinformatics toolNEBioCalculator (New England Biolbas, Ipswich, USA) (https://nebiocalculator.neb.com/#!/ssrnaamt) [22]. Because this tool only calculates this magnitude for single-stranded RNA, we multiplied the calculated result by 2.

### Evaluation of the optimal conditions and validation of RT-PCR for the IBDV-VP5 region

RT-PCR as standardized using the OneStep Ahead RT-PCR Kit (Qiagen), which has a total reaction mix volume of 25 μL, containing1X OneStep Ahead RT-PCR Master Mix (originally 2.5×, Qiagen), 1×OneStep Ahead RT-Mix (originally 25×, Qiagen), and 5 μL of viral RNA sample according to the manufacturer’s conditions and using theMastercycler proS Eppendorf thermocycler (Eppendorf, Hamburg, Germany).

Based on the mentioned concentrations, we first executed the primer annealing test by evaluating the temperature range between 49.8°C and 70°C. Then, we performed a primer concentration test evaluating the following concentrations: 1 μM, 0.7 μM, 0.5 μM, 0.25 μM, and 0.125 μM of each Farvet. VL.VP5.VL-F or Farvet. VL.VP5.VL-R primer, as recommended in a previous publication [23], (primers were diluted using PCR Grade Water from Sigma-Aldrich).

Next, we measured the detection limit in terms of the number of copies of IBDV-genomic RNA using previously prepared serial dilutions. Finally, we performed the specificity test using RNA from a second pathogenic IBDV strain (FARVET S.A.C., Peru) and nucleic acid samples from other commonly found poultry pathogens, such as infectious bronchitis virus, infectious laryngotracheitis virus, avian metapneumovirus, Newcastle disease virus, avian adenovirus responsible for inclusion body hepatitis (IBH), chicken anemia virus, and *Avibacterium paragallinarum*. In addition, whole RNA extracted from naïve embryonated eggs was used as a negative control.

Finally, the temperatures and time conditions for the conventional RT-PCR amplification of the specific product were defined as follows: Reverse transcription at 55°C for 10 min, 1 cycle of initial PCR activation step at 95°C for 5 min, 40 cycles of denaturation at 95°C for 10 s, annealing at 60°C, and extension at 72°C for 10 s, followed by a final extension step at 72°C for 2 min.

### Execution of the agarose gel electrophoresis

Ten μL of each PCR product was visualized in 2% agarose gels previously mixed 2 μL of with SYBR Safe DNA Gel Stain (Thermo Fisher Scientific). Furthermore, the electrophoretic run was executed using the O`GeneRuler DNA Ladder Mix (Sigma-Aldrich) or O’GeneRuler 50bp DNA Ladder Mix (Sigma-Aldrich) as a molecular weight marker with 1× Tris-Borate-ethylenediaminetetraacetic acid buffer (originally 10×, Sigma-Aldrich,) at 100 V for a total time of approximately 90 min.

## Results

**Figure-2:**
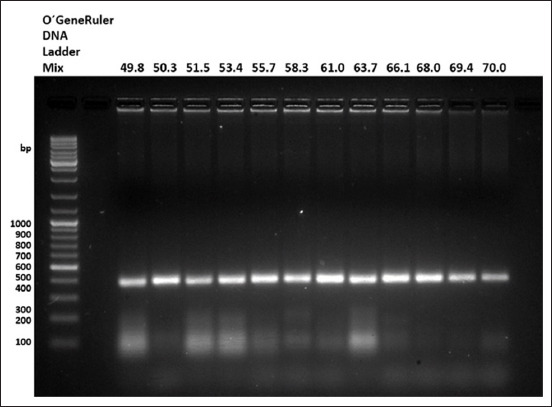
Results of the primer annealing test. A 402 bp band is consistently visible in all evaluated temperatures, but the most visible band is present in the lane of the temperature of 61°C.

The result of the *in silico* alignment of all selected IBDV VP5 genomic sequences performed with the Clustal Omega bioinformatic tool (https://www.ebi.ac.uk/Tools/msa/clustalo) produced a >405-bp consensus sequence that was very useful for selecting the specific sequence template to design our new primers (accession number AF165151.1). In addition, according to the results of the primer annealing test, a clear 402-bp band corresponding to the amplicon of interest was observed at all evaluated temperatures. However, the brightest band was observed at 61°C (Figure-2). Based on this, we selected 60°C as the definitive annealing temperature for the following tests.

**Figure-3:**
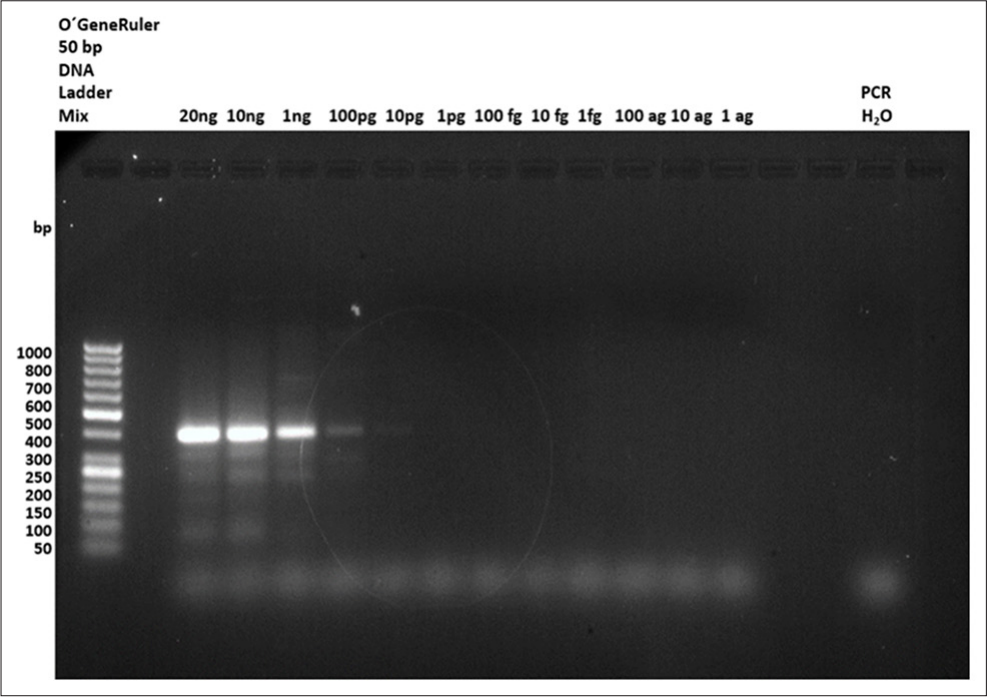
Evaluation of limit of detection test. Our newly designed primers can successfully detect bursal infectious disease virus RNA at concentrations of 0.125 M.

The limit-of-detection test results indicated that these novel primers can detect IBDV-RNA concentrations from 20 ng up to 100 pg (Figure-3). Nevertheless, the 100 pg amplicon band appeared fuzzier.

**Figure-4:**
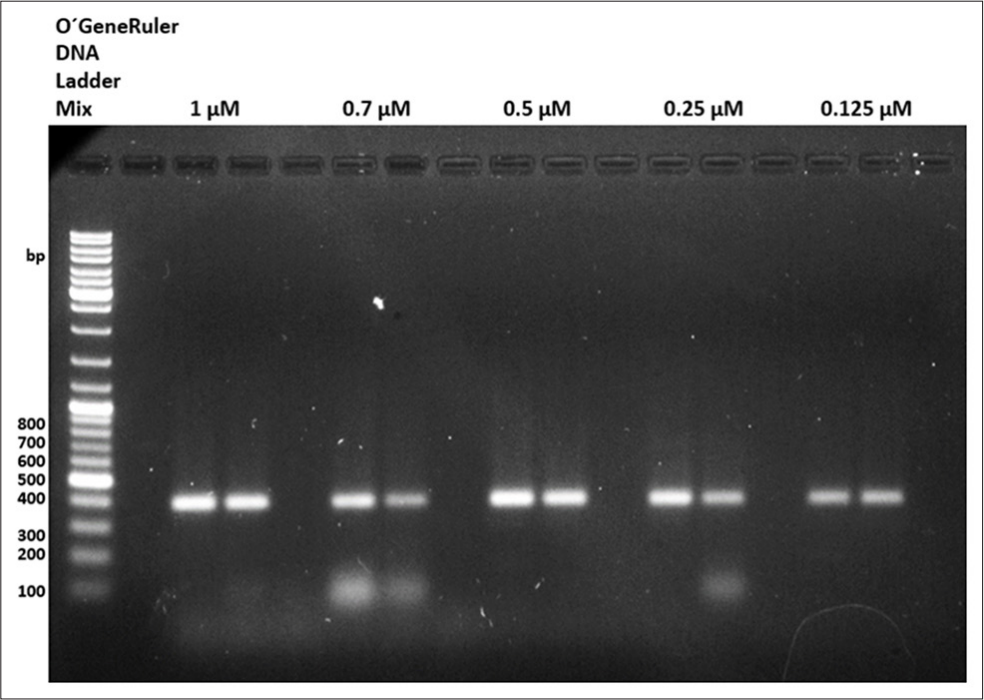
Results of the primer concentration test. Our newly designed primers can successfully detect bursal infectious disease virus RNA at a concentration of 0.125 µM.

The primer concentration test results clearly showed that we could use concentrations of these primers in a decreasing range from 1 μM to 0.125 μM (Figure-4). Nevertheless, for the final protocol of this conventional RT-PCR, we decided to apply 0.25 μM as the desired primer concentration.

**Figure-5:**
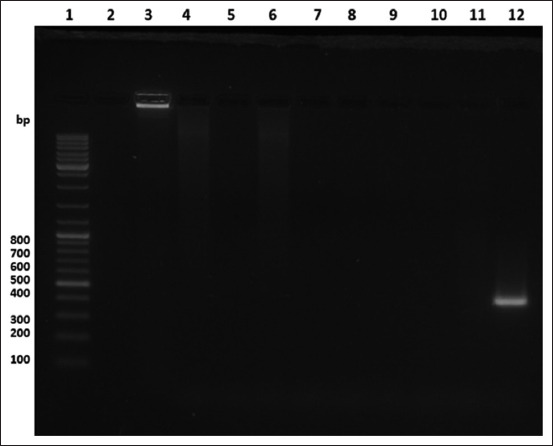
Results of specificity test. Lane 1=O’GeneRuler DNA ladder mix, Lane 2=Empty, Lane 3=Pure CEF total RNA, Lane 4=Infectious bronchitis virus positive sample, Lane 5=Infectious laryngotracheitis virus positive sample, Lane 6=Avian metapneumovirus positive sample, Lane 7=Newcastle disease virus positive sample, Lane 8=Positive sample of avian adenovirus responsible for inclusion body hepatitis. Lane 9=Chicken anemia virus-positive sample, Lane 10=Avibacterium paragallinarum-positive sample, Lane 11=PCR grade water, Lane 12=Infectious bursal disease virus-positive sample, PCR=Polymerase chain reaction.

The final test for these conventional RT-PCR was the specificity test. In this case, the results demonstrated that these novel primers exclusively amplify only the IBDV-VP5 sequence but not the chicken RNA from naïve embryonated eggs or the nucleic acid sequences of all other evaluated poultry pathogen samples (Figure-5).

## Discussion

In the present study, we focused on developing a novel and accurate conventional reverse transcriptase polymerase chain reaction (RT-PCR) procedure to specifically detect virulent variants of IBDV using newly designed primers for a consensus sequence of the gene that encodes the *VP5* protein (accession number AF165151.1). These primers were designed using a template comprising a >405-bp consensus sequence obtained from the alignment of eight different IBDV *VP5* genomic sequences. Moreover, we also demonstrated that our new RT-PCR procedure can efficiently and specifically amplify our desired IBDV sequence with just 1 ng of sample concentration of this virus RNA but not with nucleic acid samples from other commonly found poultry pathogens.

Regarding the scientific trend followed in previously published investigations on this virus, we are aware that scientific efforts have focused mainly on the development of IBDV vaccines along with the standardization of molecular techniques to detect and/or quantify this virus, targeting mainly the *VP2* sequence [3–5, 24]. However, this research trend has been gradually changing to apply other molecular techniques to target other gene sequences, such as *VP*1, *VP*3, or *VP*4, or *VP*2 along with the *VP*1 gene [4–6, 12, 25, 26]. Furthermore, molecular techniques to discriminate between virulent and non-virulent IBDV strains have been developed, and these techniques target either the *VP2* gene sequence alone or a combination of the*VP*1 and *VP*2 gene sequences [7, 12, 13, 15] or the overlapping region of *VP2* and *VP5* [16].

In this context, a scientific investigation focused on elucidating the pathogenic role of the VP5 protein in the progression of IBDV infection in juvenile chickens. This study performed experiments *in vitro*, as well as *in vivo*, with specific-pathogen-free chickens using whole IBDV-cDNA and VP5-deleted vectors and demonstrated that less exposure of juvenile chickens to VP5 counteracts its immunosuppressive effects when challenged to the avian influenza virus. This was evident when the investigators observed the reduced extent of cytopathic effects in the bursae of Fabricius juvenile chickens [27]. Méndez *et al*. [28] demonstrated that the VP5 protein plays an essential role in releasing IBD-viral particles into the extracellular milieu through non-lytic mechanisms at the early stages of infection [28].

Regarding the development of new molecular techniques for IBDV detection and/or quantitation, a newly developed one-step real-time TaqMan RT-PCR assay to discriminate between a novel variant of IBDV and other virulent and vaccine strains has been reported. This study caught our attention because the authors decided to target the VP5/VP2 overlapping region of segment A of the IBDV genome to design specific primers and TaqMan probes, resulting in efficient results [29].

Therefore, with the previously explained new paradigm for IBDV research development, we believe that it is imperative to focus scientific efforts on developing diagnostic tools targeting the consensus VP5 gene sequence of this virus, as we did with our newly designed RT-PCR procedure. In addition, we consider that our new primers represent a useful tool for future research and fieldwork to perform quick initial discrimination between the vaccine and virulent strains of IBDV present in young chicken samples from farms all over the world before executing a more costly real-time PCR procedure with or without TaqMan probes or spending an unnecessary extra budget in blindly sequencing all the samples obtained to identify only vIBDV strains.

Finally, despite our success, it is important to mention that we need to test whether these primers can also amplify the VP5 sequence of very vIBDV strains.

## Conclusion

This novel RT-PCR procedure developed in this study successfully targets and amplifies the consensus sequence of the *VP5* gene of the IBDV, ensuring high specificity for pathogenic strains. By effectively discriminating field samples containing virulent IBDV strains from non-pathogenic strains and other poultry pathogens, this method presents a cost-effective, rapid, and reliable diagnostic tool. In addtion, this approach reduces the dependency on labor-intensive and expensive sequencing techniques, facilitating quicker identification and surveillance of virulent IBDV strains in poultry populations.

Moreover, it is also important to note that while this study establishes a foundational molecular tool for IBDV diagnosis, there are several avenues for further research and application, such as: testing the RT-PCR procedure on larger field sample datasets from geographically diverse areas to validate its robustness and global applicability. Also, the incorporation of these consensus primers into a real-time RT-PCR platform may improve diagnostic speed and allow quantification of viral loads in field samples.

To sum up, by addressing these future research opportunities, this novel RT-PCR approach can be further optimized and expanded for comprehensive IBDV diagnosis, surveillance, and control strategies, ultimately contributing to improved poultry health and economic stability in the poultry industry.

## Authors’ Contributions

VL: Primer design and standardization of PCR conditions. MFD, MFS, and PO: Laboratory design of the study. MC and AM: Maintenance and replication of IBDV strains. KH: Nucleic acid extraction of samples. LT and MZ: Bioinformatic design of the study. All authors have read and approved the final manuscript.
